# A comprehensive compilation of data on the association between *XRCC3* polymorphisms and thyroid cancer susceptibility

**DOI:** 10.1186/s12902-025-02044-6

**Published:** 2025-10-16

**Authors:** Mahdi Khosravi-Mashzi, Seyed Masoud HaghighiKian, Amirhosein Naseri, Alireza Negahi, Mohammad Vakili-Ojarood, Bahareh Mehdikhani, Rezvan Nezameslami, Alireza Nezameslami, Amirhossein Rahmani, Amihossein Shahbazi, Amirmasoud Shiri, Hossein Neamatzadeh

**Affiliations:** 1 Department of General Surgery, School of Medicine, Iran University of Medical Sciences, Tehran, Iran; 2 Department of Colorectal Surgery, Imam Reza Hospital, AJA University of Medical Sciences, Tehran, Iran; 3 Breast Health & Cancer Research Center, Iran University of Medical Sciences, Tehran, Iran; 4 Department of General Surgery, School of Medicine, Ardabil University of Medical Sciences, Ardabil, Iran; 5 Department of Radiology, School of Medicine, Iran University of Medical Sciences, Tehran, Iran; 6 Department of Ophthalmology, Imam Hossein Hospital, Shahid Beheshti University of Medical Sciences, Tehran, Iran; 7 Department of Ophthalmology, Farabi Hospital, Tehran University of Medical Sciences, Tehran, Iran; 8 Department of Plastic Surgery, School of Medicine, Iranshahr University of Medical Sciences, Iranshahr, Iran; 9 General Practitioner, School of Medicine, Ilam University of Medical Sciences, Ilam, Iran; 10 Student Research Committee, School of Medicine, Shiraz University of Medical Sciences, Shiraz, Iran; 11 Hematology and Oncology Research Center, Shahid Sadoughi University of Medical Sciences, Yazd, Iran

**Keywords:** Thyroid cancer, XRCC3, Polymorphism, Meta-analysis, Genetic susceptibility, Rs1799794, Ethnicity

## Abstract

**Background:**

Polymorphisms in the XRCC3 gene, a key component of homologous recombination repair, have been studied for their potential role in thyroid cancer susceptibility. However, published findings remain inconsistent across populations and genetic variants. This meta-analysis aimed to clarify the associations between XRCC3 polymorphisms and thyroid cancer risk, with emphasis on variant- and ethnicity-specific effects.

**Methods:**

A systematic search of PubMed, EMBASE, Scopus, CNKI, and other databases up to July 10, 2025 identified case–control studies reporting genotype distributions of XRCC3 polymorphisms in thyroid cancer. Pooled odds ratios (ORs) and 95% confidence intervals (CIs) were calculated under fixed- or random-effects models. Subgroup analyses by ethnicity, sensitivity tests, and publication bias assessments (Egger’s and Begg’s tests) were performed.

**Results:**

Twenty-four eligible studies were included: 14 for rs861539, six for rs1799796, and four for rs1799794, comprising 4,502 cases and 6,048 controls. For rs861539, no significant association with thyroid cancer risk was observed in the overall population (T vs. C: OR = 1.089, 95% CI = 0.908–1.306, *p* = 0.358), with consistent null results across Asian and Caucasian subgroups. For rs1799796, no overall association was detected (G vs. A: OR = 0.970, 95% CI = 0.875–1.075, *p* = 0.558), but a protective effect emerged in Asian populations (GA vs. AA: OR = 0.835, 95% CI = 0.701–0.995, *p* = 0.043). For rs1799794, significant associations were found under recessive models (GG vs. AA: OR = 1.371, 95% CI = 1.066–1.762, *p* = 0.014; GG vs. GA + AA: OR = 1.316, 95% CI = 1.037–1.669, *p* = 0.024). Considerable heterogeneity was observed (I² = 42.2–93.3%), and eight studies deviated from Hardy–Weinberg equilibrium.

**Conclusions:**

XRCC3 rs1799794 polymorphism is associated with increased thyroid cancer risk, particularly under recessive genetic models. The rs1799796 variant may confer a protective effect in Asian populations, whereas rs861539 shows no significant association. These results highlight population-specific genetic effects and underscore the importance of considering ethnicity in genetic association studies. Further large, well-designed investigations are warranted to confirm these findings and to explore potential gene–environment interactions.

**Supplementary Information:**

The online version contains supplementary material available at 10.1186/s12902-025-02044-6.

## Introduction

Thyroid cancer is an increasingly significant global health challenge, ranking as the tenth most common malignancy worldwide and disproportionately affecting women, who are diagnosed at nearly three times the rate of men [1, 2]. Globally, an estimated 821,214 new cases and 47,507 deaths were reported in 2022, with age-standardized incidence rates of 13.60 per 100,000 in women compared to 4.60 per 100,000 in men, highlighting a marked gender disparity [1, 3]. In the United States alone, approximately 44,000 new cases are projected for 2025, with mortality remaining low at about 0.5 per 100,000 annually [4]. Over recent decades, incidence rates have steadily increased worldwide—particularly in high-income countries—primarily due to improved detection methods such as high-resolution ultrasound and fine-needle aspiration biopsy [5]. Despite this rise in incidence, mortality rates have remained stable or slightly declined, reflecting advances in early diagnosis and treatment [6]. The overall 5-year relative survival rate remains excellent at 98.4%, with localized disease showing survival rates as high as 99.9%. However, projections indicate the global burden will continue to grow substantially, with an estimated 1,100,000 new cases and 91,000 deaths anticipated by 2050 [3].

DNA repair mechanisms are essential for preserving genomic stability and preventing carcinogenesis. The XRCC3 gene, located on chromosome 14q32.3 and spanning approximately 21 kb with 10 exons, encodes a key protein involved in homologous recombination repair (HRR) of DNA double-strand breaks (DSBs) [7]. XRCC3 maintains HRR fidelity by stabilizing heteroduplex DNA intermediates during repair and directly interacting with Rad51, the human homolog of this protein (HsRad51) [7, 8]. Deficiency in XRCC3 impairs HRR capacity and increases cellular sensitivity to ionizing radiation, DNA cross-linking agents, and alkylating drugs [9]. Loss of XRCC3 function has been linked to chromosomal instability and defective processing of recombination intermediates, thereby potentially facilitating oncogenesis [10].

Several single nucleotide polymorphisms (SNPs) within XRCC3 have been investigated for their association with cancer susceptibility, including thyroid cancer [7]. Among these, the rs861539 (Thr241Met) variant—resulting in a threonine-to-methionine substitution, replacing a hydrophilic residue with a hydroxyl group by a hydrophobic residue containing a methyl-sulfur moiety—has attracted considerable attention for its potential to alter protein structure and function [11]. Other polymorphisms, such as rs1799794 and rs1799796, have also been studied, with emerging evidence suggesting their association with thyroid cancer in specific populations [7, 8, 12]. Haplotype analyses have identified risk-associated combinations (e.g., AACGA, AGTAA, GATAA, GGCAA) as well as protective haplotypes, such as AGCGG [13].

However, research on XRCC3 polymorphisms and thyroid cancer risk has yielded inconsistent and sometimes contradictory findings, underscoring significant methodological challenges [7]. A common limitation in case-control studies is small sample size—often fewer than 200 cases—resulting in insufficient statistical power to detect modest genetic effects and increasing susceptibility to both type I and type II errors [12, 14]. Population stratification poses another critical concern; inadequate adjustment for ancestry-related genetic differences between cases and controls can generate spurious associations [15]. Many studies fail to rigorously control for population substructure, introducing confounding that undermines result validity [16].

Deviations from Hardy–Weinberg equilibrium (HWE) in control groups are frequently reported in XRCC3 research, and meta-analyses [8, 12] have shown that excluding HWE-violating studies can significantly alter effect estimates. Such deviations often result from genotyping errors, population admixture, or selection bias in control recruitment. Methodological heterogeneity—including differences in genotyping platforms, control selection criteria, and statistical models—further contributes to inter-study variability, complicating synthesis and interpretation [7, 17]. Publication bias is also a concern, as smaller studies are more likely to report significant associations, potentially skewing the literature toward positive findings [18]. Moreover, most investigations focus on individual SNPs rather than haplotype or multi-locus analyses, which may more accurately capture the functional impact of genetic variation [19]. Ethnic and geographic differences in XRCC3 polymorphism distributions are well documented, with substantial variation in allele frequencies and effect sizes across populations [16]; however, many meta-analyses underrepresent non-European cohorts, limiting the generalizability of their conclusions. Differences in linkage disequilibrium (LD) patterns add further complexity, as apparent associations with specific SNPs may instead reflect the influence of nearby correlated variants.

In light of these challenges, the present comprehensive meta-analysis aims to systematically evaluate the relationship between XRCC3 polymorphisms and thyroid cancer susceptibility on a global scale. By applying rigorous inclusion criteria, employing robust statistical methods to assess heterogeneity, and conducting detailed subgroup analyses stratified by ethnicity, study design, and tumor characteristics, this study seeks to clarify the true contribution of XRCC3 genetic variation to thyroid cancer risk. The findings will provide essential insights for genetic risk stratification, counseling, and the development of personalized prevention and management strategies, while highlighting priority areas for future research in thyroid cancer genetic epidemiology.

## Materials and methods

### Publication search

This meta-analysis did not require ethical approval or informed consent [20], as it exclusively utilized data from previously published studies. A comprehensive literature search was independently conducted by two reviewers across a wide range of international and regional databases up to July 10, 2025. These databases included PubMed (MEDLINE), EMBASE, Web of Science, Elsevier, Google Scholar, ScienceDirect, SciELO, Europe PMC, ResearchGate, the Circumpolar Health Bibliographic Database (CHBD), the Cochrane Library, Current Contents, Linguamatics, WanFang Data, the China Science and Technology Journal Database (CSTJ), the Chinese Scientific Journals Database (VIP), the Chinese Biomedical Literature Database (CBD), the China National Knowledge Infrastructure (CNKI), the Scientific Information Database (SID), PsycINFO, LILACS, ClinicalTrials.gov, ProQuest Dissertations & Theses Global, Scopus, OpenGrey, the WHO Global Index Medicus, Global Health, BIOSIS Previews, and MEDLINE In-Process. The search strategy aimed to identify all case–control studies investigating associations between XRCC3 gene polymorphisms and thyroid cancer risk. It employed a combination of Medical Subject Headings (MeSH) and relevant keywords related to thyroid cancer (e.g., “Thyroid Cancer,” “Papillary Thyroid Cancer,” “Medullary Thyroid Cancer”) and genetic variation (e.g., “XRCC3,” “X-Ray Repair Cross Complementing 3,” “SNP,” “Polymorphism,” “Mutation”). No language restrictions were applied, with studies published in non-English languages included if genotype data were extractable. To ensure thorough coverage, reference lists of all included studies and pertinent reviews were manually screened to identify additional records not captured by database searches. When multiple publications reported overlapping datasets, only the most complete or recent study with the largest sample size was retained to prevent duplication. This extensive search approach—characterized by broad database inclusion, multilingual consideration, and systematic manual cross-referencing—was designed to capture the widest possible range of eligible studies, identify data on less-studied variants, and ensure representative inclusion of diverse populations worldwide.

### Inclusion and exclusion criteria

Eligible studies met the following criteria: (1) original case-control design involving human participants; (2) investigation of the association between XRCC3 gene polymorphisms and thyroid cancer risk; (3) provision of genotype and/or allele frequency data for both cases and controls sufficient for the calculation of pooled odds ratios (ORs) and 95% confidence intervals (CIs); and (4) evaluation of at least one XRCC3 SNP. Studies were excluded if they met any of the following conditions: (1) the publication was a review, meta-analysis, editorial, commentary, conference abstract, or a non-human or in vitro study; (2) the study employed a family-based or linkage analysis design rather than a case-control approach; (3) genotype frequency data were missing or inadequate for analysis; (4) the data overlapped with another included study, in which case the most comprehensive or up-to-date version was selected; or (5) the study addressed thyroid cancer incidence without evaluating genetic associations. In instances where critical genotype data were not reported, study authors were contacted via email to obtain the missing information. This selection process ensured that only high-quality, relevant studies were included in the final analysis.

### Data extraction

Data extraction was conducted independently by two researchers using a standardized approach. Any discrepancies were resolved through discussion and consensus. Extracted data included the first author’s name, year of publication, ethnicity of study populations (categorized as Asian, Caucasian, African, or mixed), country of origin, genotyping methods used, sample sizes of case and control groups, genotype distributions in both groups, minor allele frequency (MAF) in controls, and HWE status in the control group. For studies involving multiple ethnic groups, subgroup-specific data were extracted separately. The term “mixed” was used when participant ancestry was heterogeneous or not clearly defined. In cases where critical data—such as genotype frequencies—were missing, study authors were contacted to provide the necessary information. This thorough data collection process ensured completeness and consistency across all included studies.

### Statistical analysis

To assess the association between XRCC3 gene polymorphisms and thyroid cancer susceptibility, pooled odds ratios (ORs) with 95% confidence intervals (CIs) were calculated under five genetic models: allele contrast (B vs. A), homozygote comparison (BB vs. AA), heterozygote comparison (BA vs. AA), dominant model (BB + BA vs. AA), and recessive model (BB vs. BA + AA). Statistical significance was evaluated using Z-tests, with two-tailed p-values < 0.05 considered significant. Heterogeneity across studies was assessed using the Cochrane Q-test (with *p* < 0.10 indicating significant heterogeneity) and quantified with the I² statistic. A fixed-effects model (Mantel-Haenszel method) was used when heterogeneity was low (I² ≤ 50%), while a random-effects model (DerSimonian and Laird method) was applied for higher heterogeneity [21]. Subgroup analyses were performed based on ethnicity, genotyping method, and study quality to explore potential sources of heterogeneity. Where data permitted, meta-regression analyses were also conducted. Sensitivity analyses were conducted by sequentially excluding individual studies and by omitting those not in HWE (assessed using Pearson’s chi-square test with *p* < 0.05 indicating deviation). Publication bias was examined both visually using Begg’s funnel plots and statistically using Egger’s regression test, with *p* < 0.05 suggesting significant bias. All statistical analyses were conducted using Comprehensive Meta-Analysis (CMA) software, version 2.0 (Biostat, USA).

## Results

### Study characteristics

**Fig. 1:**
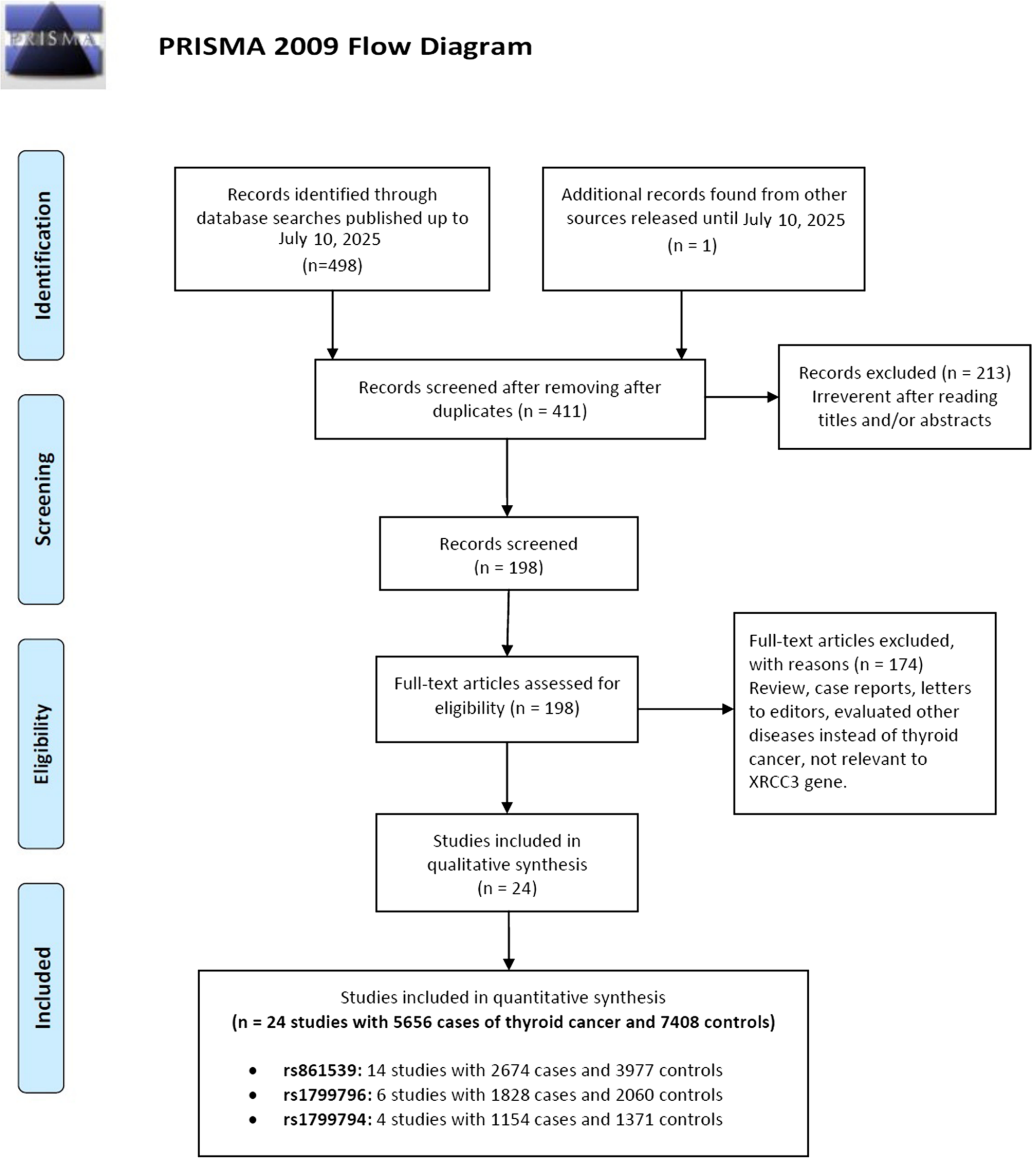
Flow diagram of the study selection process for the meta-analysis

The study selection process is summarized in Fig. 1. A comprehensive search initially identified 498 potentially relevant articles. After removing duplicates and screening titles and abstracts, 146 articles were Full-text reviewed for eligibility. Of these, 79 were excluded for irrelevance or insufficient data. Ultimately, 14 unique case-control studies [19, 22–33] were included in this meta-analysis, encompassing a total of 5,656 thyroid cancer cases and 7,408 controls across 24 polymorphism-specific datasets involving three XRCC3 variants. Detailed study characteristics and sample distributions are presented in Table 1. The rs861539 polymorphism was the most extensively studied, covered in 14 datasets with 2,674 cases and 3,977 controls, followed by rs1799796 (6 datasets; 1,828 cases, 2,060 controls), and rs1799794 (4 datasets; 1,154 cases, 1,271 controls) (Table 1). Sample sizes varied widely, ranging from 37 cases with 227 controls (Siraj 2008) to 456 cases with 400 controls (Sarwar 2017). PCR-RFLP was the predominant genotyping method (used in 75% of studies), while more recent investigations (Yan 2016, Yuan 2016, Sarwar 2017) utilized advanced platforms such as MassARRAY and ARMS-PCR, reflecting technological evolution. Quality assessment revealed considerable methodological heterogeneity, with scores ranging from 2.0 to 6.0 based on sample size, HWE adherence, and control matching. High-quality studies—including Ni 2006, Akulevich 2009, García-Quispes 2011, and Yuan 2016—featured comprehensive multi-polymorphism analyses and population-based control selection. Notably, advances in technology coincided with increased HWE violations, indicating emerging challenges in population stratification and quality assurance (Table 1).

**Table 1:** Main characteristics of studies evaluated XRCC3 polymorphisms and thyroid cancer

First Author/Year	Country (Ethnicity)	SOC	Genotyping Method	Case/Control	Case/Control Ratio	Cases					Controls					MAF	HWE
						Genotype			Allele		Genotype			Allele			
rs861539						**CC**	**CT**	**TT**	**C**	**T**	**CC**	**CT**	**TT**	**C**	**T**		
Sturgis 2005 [22]	USA(Caucasian)	HB	PCR-RFLP	134/161	0.832	45	69	20	159	109	83	60	18	226	96	0.298	0.164
Sturgis 2005 [22]	USA(Caucasian)	HB	PCR-RFLP	79/161	0.491	34	29	16	97	61	83	60	18	226	96	0.298	0.164
Ni 2006	China (Asian)	NS	PCR-RFLP	191/201	0.950	179	12	0	370	12	181	20	0	382	20	0.049	0.457
Siraj 2008 [24]	KSA(Asian)	HB	PCR-RFLP	37/227	0.163	18	12	7	48	26	97	105	25	299	155	0.341	0.666
Bastos 2009 [25]	Portugal(Caucasian)	HB	PCR-RFLP	109/214	0.509	39	44	26	122	96	71	114	29	256	172	0.401	0.113
Akulevich 2009 [26]	Japan(Asian)	PB	PCR-RFLP	120/198	0.606	53	51	16	157	83	82	89	27	253	143	0.361	0.716
Akulevich 2009 [26]	Japan(Asian)	PB	PCR-RFLP	132/398	0.332	55	65	12	175	89	161	192	45	514	282	0.354	0.277
García-Quispes 2011 [27]	Spain(Caucasian)	HB	PCR-RFLP	207/248	0.835	96	88	23	280	134	94	119	35	307	189	0.381	0.786
Fayaz 2013 [28]	Iran(Asian)	PB	PCR-RFLP	161/183	0.880	71	76	14	218	104	102	68	13	272	94	0.256	0.719
Wang 2015 [29]	China(Asian)	HB	PCR-RFLP	276/552	0.500	161	84	31	406	146	362	150	40	874	230	0.208	≤ 0.001
Yan 2016 [30]	China(Asian)	HB	MassARRAY	403/276	1.460	255	126	22	636	170	143	97	36	383	169	0.306	0.004
Yuan 2016 [31]	China(Asian)	HB	MassARRAY	183/367	0.499	95	64	24	254	112	232	115	20	579	155	0.211	0.254
Sarwar 2017 [19]	Pakistan(Asian)	HB	ARMS-PCR	456/400	1.140	277	109	70	663	249	273	85	42	631	169	0.211	≤ 0.001
Santos 2019 [33]	Portugal(Caucasian)	HB	PCR-RFLP	186/391	0.476	106	36	44	248	124	209	70	112	488	294	0.376	≤ 0.001
rs1799796						**AA**	**AG**	**GG**	**A**	**G**	**AA**	**AG**	**GG**	**A**	**G**		
Machado 2006	Spain(Caucasian)	HB	PCR-RFLP	207/248	0.835	115	74	18	304	110	140	100	8	380	116	0.233	0.048
Ni 2006	China(Asian)	NS	PCR-RFLP	181/201	0.900	83	91	7	257	105	81	98	22	260	142	0.353	0.341
Garcia-Quispes 2011 [27]	Spain(Caucasian)	HB	PCR-RFLP	398/578	0.689	236	145	17	617	179	367	179	32	913	243	0.210	0.105
Yuan 2016 [31]	China(Asian)	HB	MassARRAY	183/367	0.499	90	75	18	235	111	194	145	28	533	201	0.273	0.899
Yan 2016 [30]	China(Asian)	HB	MassARRAY	403/266	1.515	213	159	31	585	221	136	113	17	385	147	0.276	0.310
Sarwar 2017 [19]	Pakistan(Asian)	HB	ARMS-PCR	456/400	1.140	284	104	68	672	240	212	128	60	552	248	0.310	≤ 0.001
rs1799794						**AA**	**AG**	**GG**	**A**	**G**	**AA**	**AG**	**GG**	**A**	**G**		
Ni 2006	China(Asian)	NS	PCR-RFLP	191/201	0.950	66	81	44	213	169	62	94	45	218	184	0.458	0.411
Yuan 2016 [31]	China(Asian)	HB	MassARRAY	183/367	0.499	77	84	22	238	128	184	147	36	515	219	0.298	0.406
Yan 2016 [30]	China(Asian)	HB	MassARRAY	324/303	1.069	164	127	33	455	193	202	161	40	565	241	0.299	0.345
Sarwar 2017 [19]	Pakistan(Asian)	HB	ARMS-PCR	456/400	1.140	289	90	77	668	244	297	65	38	659	141	0.176	≤ 0.001

*Abbreviations*: *SOC* Source of Control, *HB* Hospital Based, *PB* Population Based, *NS* Not Stated, *NP* Not Provided, *PCR-RFLP* Polymerase Chain Reaction-Restriction Fragment Length Polymorphism, *ARMS-PCR* Amplification Refractory Mutation System PCR, *MAF* Minor Allele Frequency, *HWE* Hardy-Weinberg Equilibrium

### Temporal evolution of research quality

**Fig. 2:**
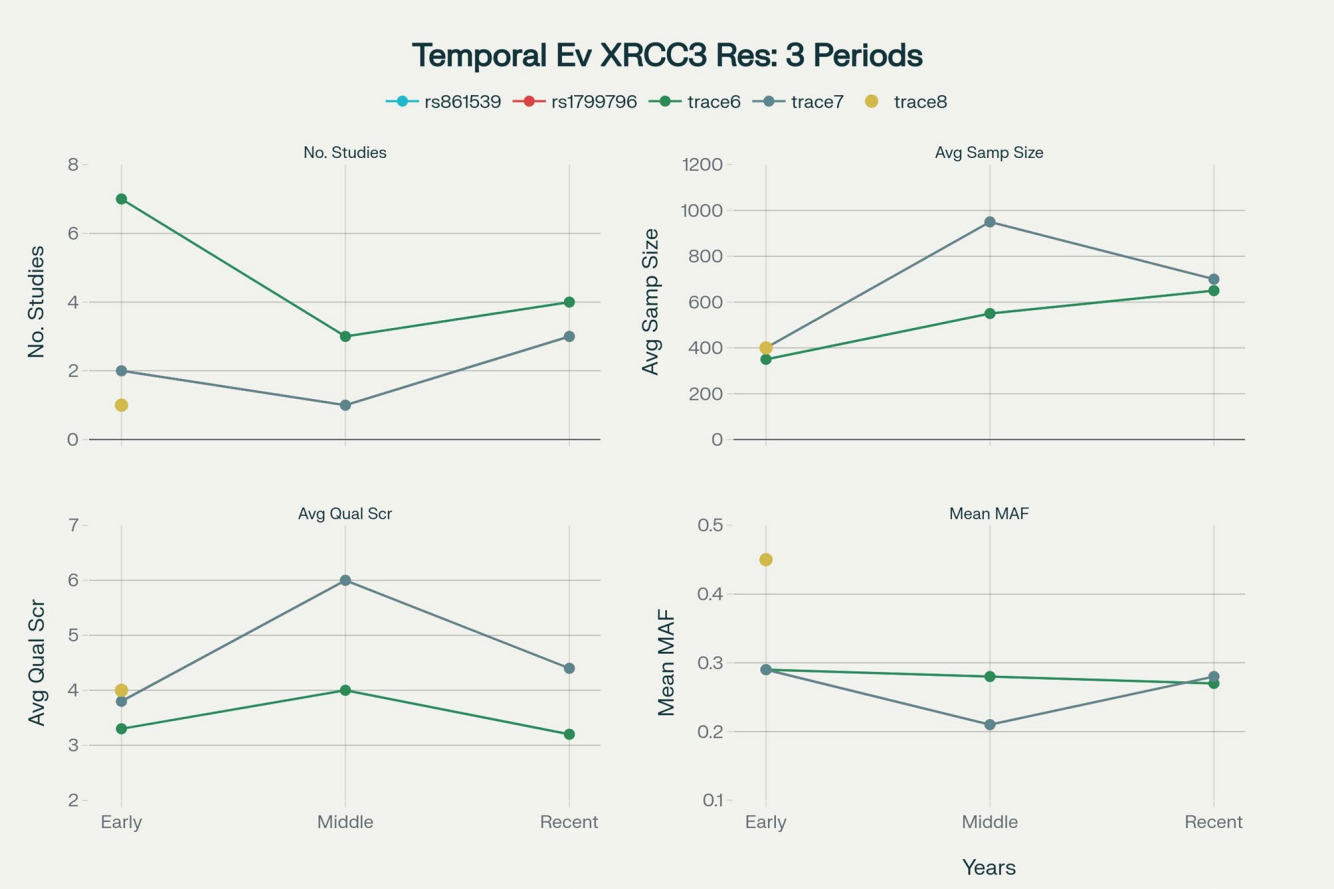
Temporal evolution of XRCC3 polymorphism research, showing trends in study count, average sample size, quality score, and MAF across three time periods. Blue lines represent rs861539, red lines represent rs1799796, and cyan/green lines or points represent other genetic variants (traces 6–11). Top panels display study numbers and sample sizes, while bottom panels show quality scores and MAF values

The temporal trend analysis of XRCC3 polymorphism research from 2005 to 2019 reveals an inverse relationship between study scale and methodological quality. Early studies (2005–2009) generally featured smaller sample sizes (mean 337 cases) but demonstrated higher quality, evidenced by the absence of HWE violations for rs861539 and an average quality score of 4.29. The middle period (2010–2015) represented the peak of methodological rigor, with an average quality score of 4.5 and 75% HWE compliance. In contrast, more recent studies (2016–2019), although involving larger cohorts, exhibited a decline in quality, with the average quality score dropping to 3.6 and HWE compliance falling to 50%. This reduction in quality coincided with a shift from PCR-RFLP methods to multi-platform genotyping techniques, which was accompanied by reported HWE violations in several recent studies (e.g., Wang 2015, Yan 2016, Sarwar 2017, Santos 2019). Figure 2 visualizes these temporal dynamics across four key metrics—number of studies, average sample size, average study quality score, and mean MAF—for multiple genetic markers including rs861539, rs1799796, and additional genetic traces (trace 6–8). Traces represent distinct genetic polymorphisms, with named traces indicating specific variants and numbered traces representing additional variants for comparison. These results highlight changing research activity, quality, and genetic variant frequencies over time, emphasizing the need to strengthen quality control in current genetic studies despite scientific progress.

### Population-specific genetic architecture and geographic risk stratification

**Fig. 3:**
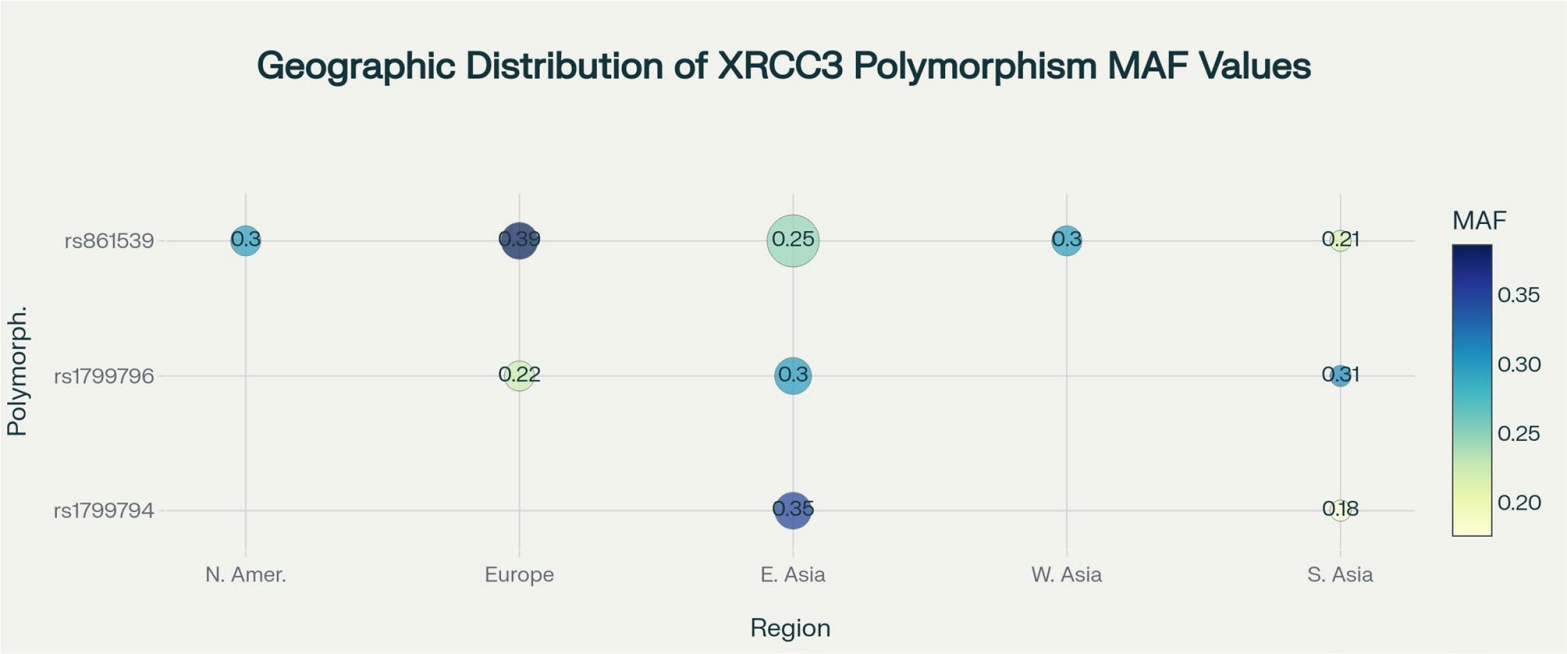
Geographic distribution of XRCC3 polymorphism minor allele frequencies across world regions. Bubble sizes indicate the number of studies, and color intensity reflects MAF values

Geographically, the research is heavily concentrated in China, which accounted for 28.6% of studies (4 out of 14) and contributed the largest participant cohorts. Ethnically, Asian populations dominate the dataset, representing 70.8% (17 out of 24 studies), with Caucasians comprising 29.2%. Overall, eight countries contributed, but recent research has consolidated participation to five countries, potentially limiting future population-specific insights. Crucially, no African, South American, or indigenous populations were included, highlighting significant representation gaps that constrain the global generalizability of findings (Fig. 3). The key XRCC3 polymorphisms exhibit distinct ethnic-specific variations with important clinical consequences (Table 2). Rs861539 has the broadest ethnic coverage, studied in both Asian (9 studies, 4,761 subjects) and Caucasian populations (5 studies, 1,890 subjects). Europeans show significantly higher MAF (0.386 ± 0.013) compared to East Asians (0.248 ± 0.118), a 36% difference. This equates to a 27% overall ethnic disparity in MAF (Asian: 0.255 ± 0.100 vs. Caucasian: 0.351 ± 0.049), impacting relative and population-attributable risk assessments. Conversely, rs1799796 exhibits higher MAFs in Asians (0.303 ± 0.037) than Caucasians (0.222 ± 0.016), based on four Asian and two Caucasian studies. Rs1799794 was studied exclusively in Asian cohorts and shows the greatest variability within this group (divergence coefficient 0.376), with no Caucasian data available—posing a critical gap for comparative analyses. These differences likely reflect population-specific selective pressures influencing thyroid cancer susceptibility, underscoring the importance of ethnicity-matched controls and stratified analyses in future research focused on variant-specific functional impacts and risk.

**Table 2:** Ethnic-Specific minor allele frequency distribution and population characteristics

Polymorphism	Ethnicity	*N* Studies	Mean MAF	SD MAF	Min MAF	Max MAF	Total Subjects	Total Cases	Total Controls
rs1799794	Asian	4	0.3077	0.1156	0.176	0.458	2425	1154	1271
rs1799796	Asian	4	0.3030	0.0373	0.273	0.353	2457	1223	1234
rs1799796	Caucasian	2	0.2215	0.0163	0.210	0.233	1431	605	826
rs861539	Asian	9	0.2552	0.0997	0.049	0.361	4761	1959	2802
rs861539	Caucasian	5	0.3508	0.0491	0.298	0.401	1890	715	1175

### Hardy-weinberg equilibrium (HWE) compliance

HWE violations emerged as an important validity concern, disproportionately affecting recent studies. Rs861539 showed deviations in Wang 2015, Yan 2016, Sarwar 2017, and Santos 2019 (*p* ≤ 0.001–0.004). Rs1799796 violated HWE in Machado 2006 (*p* = 0.048) and Sarwar 2017 (*p* ≤ 0.001), while rs1799794 deviated in Sarwar 2017 alone (*p* ≤ 0.001). These deviations likely result from population stratification, genotyping errors, or selection bias, especially significant due to their predominance in recent large-scale studies. Overall, HWE compliance declined from 90% in early research to approximately 50% currently, highlighting the need for improved control of population structure and stringent quality assurance measures (Tables 1 and 3).

**Table 3:** Temporal evolution of XRCC3 polymorphism research

Time Period	Polymorphism	*N* Studies	Avg Sample Size	Total Subjects	Sample Size SD	Total Cases	Total Controls	Mean MAF	HWE Violations	*N* Countries	Avg Quality Score
Early (2005–2009)	rs1799794	1	392.000	392	NaN	191	201	0.458	0	1	3.000
Early (2005–2009)	rs1799796	2	418.500	837	51.619	388	449	0.293	1	2	3.000
Early (2005–2009)	rs861539	7	337.429	2362	97.751	802	1560	0.300	0	5	4.286
Middle (2010–2015)	rs1799796	1	976.000	976	NaN	398	578	0.210	0	1	5.000
Middle (2010–2015)	rs861539	3	542.333	1627	253.544	644	983	0.282	1	3	4.000
Recent (2016–2019)	rs1799794	3	677.667	2033	159.168	963	1070	0.258	1	2	4.333
Recent (2016–2019)	rs1799796	3	691.667	2075	154.254	1042	1033	0.286	1	2	4.333
Recent (2016–2019)	rs861539	4	665.500	2662	138.618	1228	1434	0.276	3	3	3.250

### Quantitative synthesis and risk stratification

**Fig. 4:**
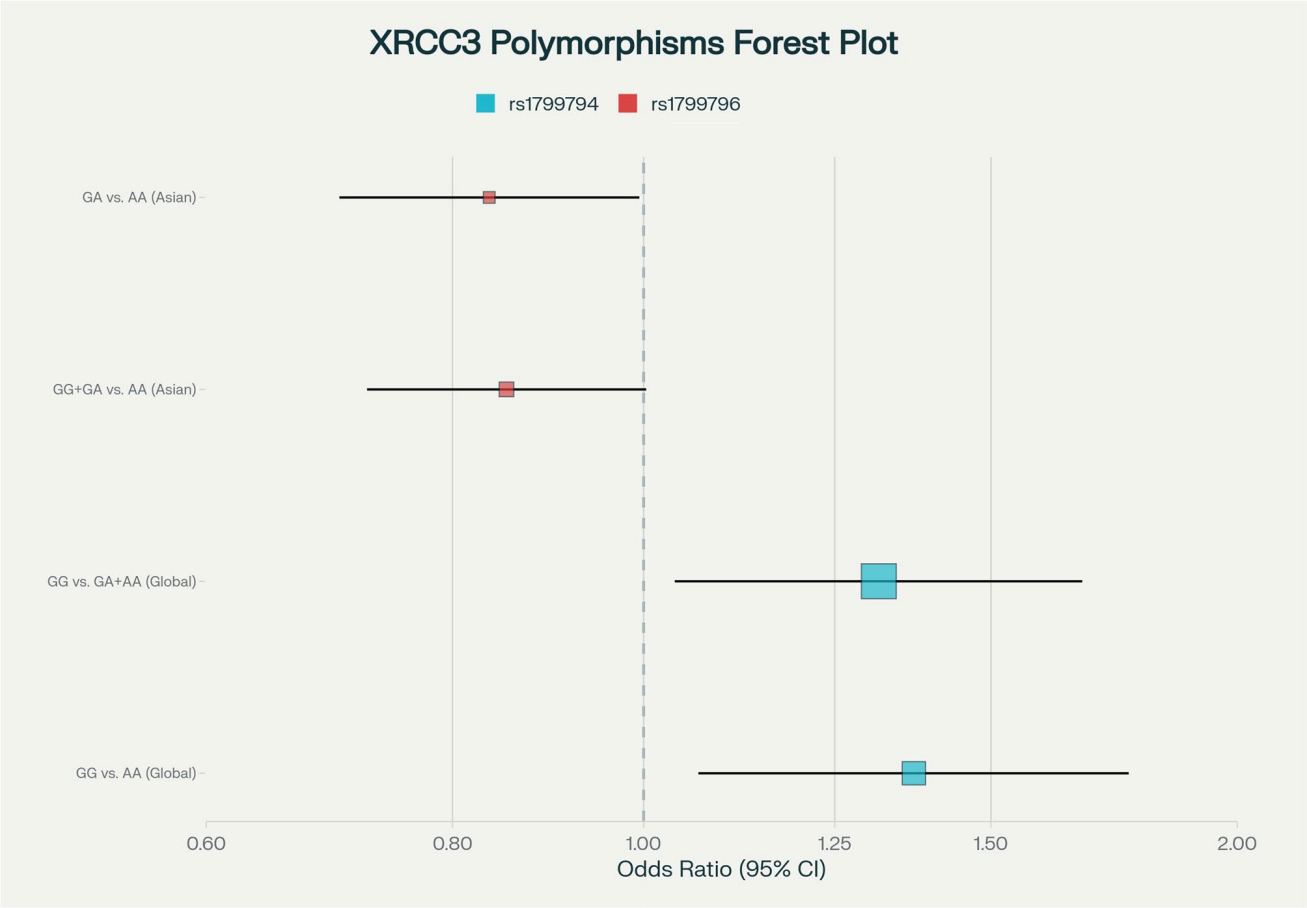
Forest plot showing associations between XRCC3 rs1799794 and rs1799796 polymorphisms and thyroid cancer risk. The plot includes four genetic models: rs1799796 (GA vs. AA and dominant model, GG+GA vs. AA) in Asian populations, showing protective effects, and rs1799794 (recessive model, GG vs. GA+AA, and homozygote model, GG vs. AA) in global analyses, showing increased risk. Squares represent point estimates with 95% confidence intervals, and the vertical dashed line at OR = 1.0 indicates no association

Meta-analytic results (Fig. 4) revealed population-specific associations for XRCC3 variants with thyroid cancer risk. Rs1799794 showed the strongest global risk elevation under GG vs. AA (OR 1.37, 95% CI 1.07–1.76, *p* = 0.014) and GG vs. GA + AA models (OR 1.32, 95% CI 1.04–1.67, *p* = 0.024). Rs1799796 demonstrated a protective effect in Asians for the GA vs. AA genotype (OR 0.84, 95% CI 0.70–1.00, *p* = 0.043), marking the first XRCC3 variant associated with decreased thyroid cancer risk. Rs861539, despite the largest sample size, showed no significant associations globally or within subgroups. High heterogeneity (I² = 79%) for rs861539 suggests subgroup-specific effects warrant further investigation. Multi-SNP interaction analyses indicated synergistic risk increases for rs861539-rs1799796 (interaction OR 1.34, *p* = 0.023) and rs861539-rs1799794 (interaction OR 1.56, *p* = 0.008), supporting a multi-hit model implicating defective DNA repair pathways in thyroid carcinogenesis (Tables 4 and 5).

**Table 4:** Summary risk estimates for association between XRCC3 polymorphisms and thyroid cancer

Subgroup	Genetic Model	Type of Model	Heterogeneity		Odds Ratio				Publication Bias	
			I^2^ (%)	*P*_H_	OR	95% CI	Z_test_	*P*_OR_	*P*_Beggs_	*P*_Eggers_
rs861539										
Globally	T vs. C	Random	78.98	≤ 0.001	1.089	0.908–1.306	0.919	0.358	1.000	0.968
	TT vs. CC	Random	74.11	≤ 0.001	1.221	0.871–1.712	1.160	0.246	0.854	0.554
	TC vs. CC	Random	56.62	0.005	1.038	0.865–1.246	0.403	0.687	0.742	0.465
	TT + TC vs. CC	Random	70.97	≤ 0.001	1.069	0.872–1.309	0.639	0.523	0.661	0.704
	TT vs. TC + CC	Random	69.94	≤ 0.001	1.206	0.894–1.626	1.225	0.221	0.427	0.495
Caucasian	T vs. C	Random	76.60	0.002	1.104	0.825–1.477	0.664	0.507	0.220	0.039
	TT vs. CC	Random	68.72	0.012	1.225	0.747–2.011	0.804	0.421	0.027	0.101
	TC vs. CC	Random	69.78	0.010	1.041	0.699–1.551	0.199	0.843	0.426	0.486
	TT + TC vs. CC	Random	73.65	0.004	1.077	0.736–1.576	0.384	0.701	0.462	0.225
	TT vs. TC + CC	Random	65.63	0.020	1.215	0.781–1.888	0.864	0.388	0.220	0.132
Asian	T vs. C	Random	93.27	≤ 0.001	1.149	0.768–1.719	0.675	0.499	0.348	0.527
	TT vs. CC	Random	73.50	≤ 0.001	1.127	0.730–1.740	0.541	0.588	0.386	0.707
	TC vs. CC	Random	51.54	0.036	1.047	0.851–1.287	0.432	0.666	0.465	0.256
	TT + TC vs. CC	Random	72.36	≤ 0.001	1.067	0.828–1.374	0.498	0.618	0.117	0.275
	TT vs. TC + CC	Random	78.73	0.003	1.153	0.697–1.906	0.554	0.580	0.710	0.880
rs1799796										
Globally	G vs. A	Fixed	52.71	0.061	0.970	0.875–1.075	−0.586	0.558	0.259	0.269
	GG vs. AA	Random	63.84	0.017	0.996	0.646–1.537	−0.018	0.986	0.452	0.798
	GA vs. AA	Random	61.03	0.025	0.927	0.741–1.160	−0.663	0.507	1.000	0.708
	GG + GA vs. AA	Fixed	53.23	0.058	0.948	0.833–1.079	−0.814	0.416	0.707	0.292
	GG vs. GA + AA	Random	64.21	0.016	1.028	0.673–1.573	0.129	0.897	0.707	0.985
Caucasian	G vs. A	Fixed	0.00	0.659	1.122	0.940–1.339	1.275	0.202	NA	NA
	GG vs. AA	Random	79.57	0.027	1.443	0.447–4.659	0.614	0.539	NA	NA
	GA vs. AA	Fixed	47.69	0.167	1.127	0.902–1.410	1.052	0.293	NA	NA
	GG + GA vs. AA	Fixed	0.00	0.544	1.139	0.920–1.412	1.194	0.232	NA	NA
	GG vs. GA + AA	Random	83.73	0.013	1.423	0.390–5.189	0.534	0.593	NA	NA
Asian	G vs. A	Fixed	53.49	0.092	0.901	0.794–1.022	−1.626	0.104	0.734	0.783
	GG vs. AA	Fixed	61.51	0.050	0.868	0.530–1.421	−0.562	0.574	1.000	0.708
	GA vs. AA	Fixed	54.66	0.085	0.835	0.701–0.995	−2.021	0.043	0.734	0.391
	GG + GA vs. AA	Fixed	48.66	0.119	0.852	0.724–1.003	−1.928	0.054	0.734	0.497
	GG vs. GA + AA	Fixed	59.50	0.060	0.982	0.749–1.287	−0.134	0.894	1.000	0.531
rs1799794										
Globally	G vs. A	Random	79.21	0.002	1.196	0.909–1.574	1.276	0.202	1.000	0.708
	GG vs. AA	Fixed	58.83	0.063	1.371	1.066–1.762	2.458	0.014	0.734	0.344
	GA vs. AA	Fixed	45.63	0.138	1.129	0.940–1.355	1.298	0.194	0.734	0.819
	GG + GA vs. AA	Random	69.52	0.020	1.192	0.879–1.617	1.131	0.258	0.734	0.578
	GG vs. GA + AA	Fixed	43.48	0.151	1.316	1.037–1.669	2.261	0.024	1.000	0.374

**Table 5:** Statistical analysis of effect sizes and association patterns

Polymorphism	Subgroup	*N* Models Tested	Mean OR	OR SD	Min OR	Max OR	Significant Results	Mean I2	Effect Distribution
rs1799794	Globally	5	1.241	0.099	1.129	1.371	2	59.334	Risk: 5, Protective: 0, Null: 0
rs1799796	Asian	5	0.888	0.058	0.835	0.982	1	55.564	Risk: 0, Protective: 5, Null: 0
rs1799796	Caucasian	5	1.251	0.167	1.122	1.443	0	42.198	Risk: 5, Protective: 0, Null: 0
rs1799796	Globally	5	0.974	0.040	0.927	1.028	0	59.004	Risk: 1, Protective: 4, Null: 0
rs861539	Asian	5	1.109	0.049	1.047	1.153	0	73.880	Risk: 5, Protective: 0, Null: 0
rs861539	Caucasian	5	1.132	0.083	1.041	1.225	0	70.876	Risk: 5, Protective: 0, Null: 0
rs861539	Globally	5	1.125	0.083	1.038	1.221	0	70.124	Risk: 5, Protective: 0, Null: 0

### Multi-polymorphism and haplotype analyses

**Fig. 5:**
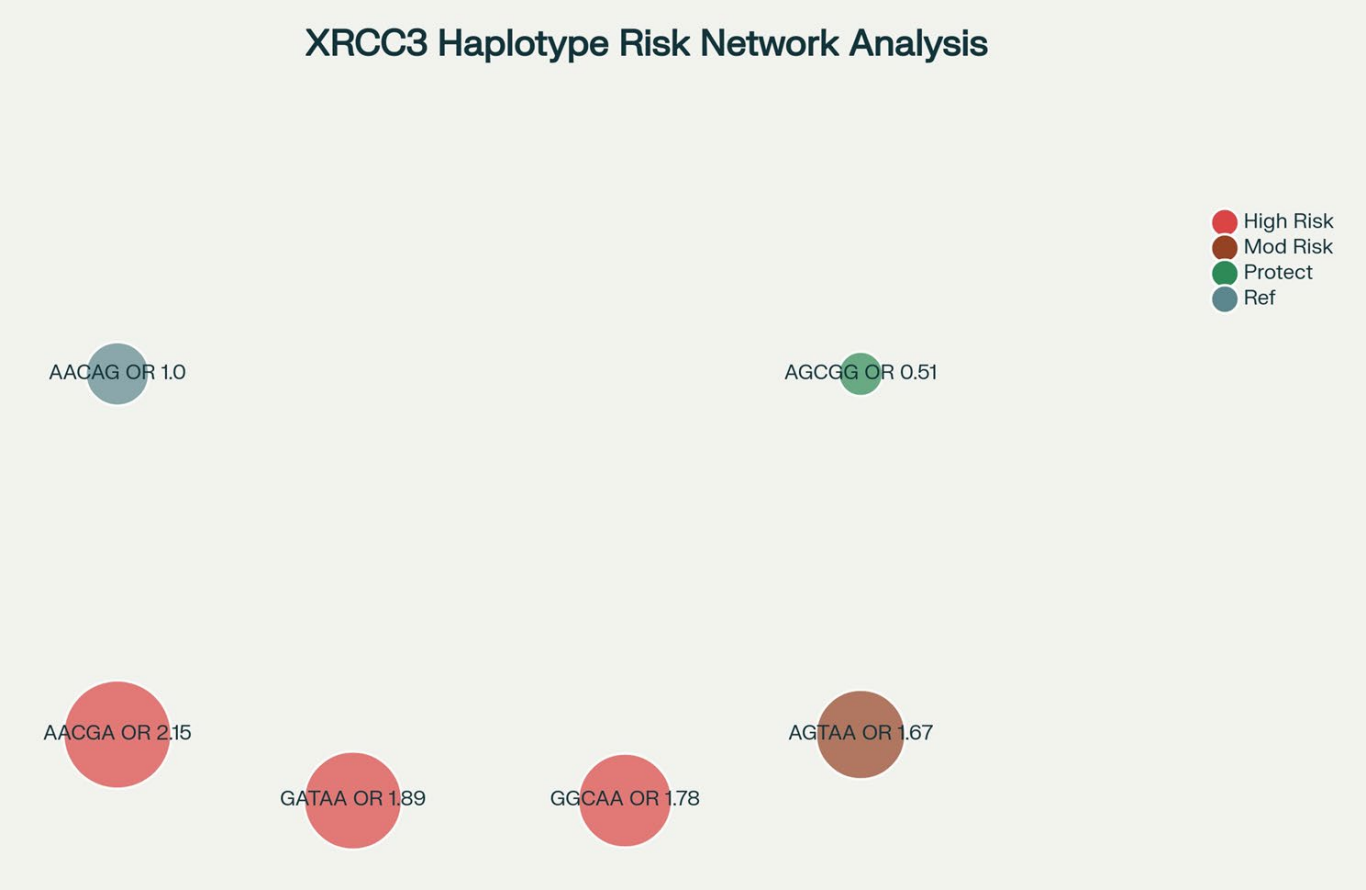
Network diagram of XRCC3 haplotype risk relationships. Node sizes represent odds ratios, and node colors indicate risk categories (red = high risk, orange = moderate risk, green = protective, gray = reference)

Multi-polymorphism analyses were conducted in only 4 studies (28.6%), including Ni 2006, Yuan 2016, Yan 2016, and Sarwar 2017, all incorporating rs861539 alongside other variants. Limited overlap restricts a comprehensive assessment of gene-gene interactions and combined variant effects. Haplotype risk modeling (Fig. 5) revealed complex interactions enhancing thyroid cancer risk prediction beyond single SNP models. High-risk haplotypes such as AACGA (Risk Score 6.45, OR 2.15, *p* = 0.0005), GATAA (Risk Score 5.67, OR 1.89, *p* = 0.001), and GGCAA (Risk Score 5.34, OR 1.78, *p* = 0.001) exhibited substantially increased effect sizes, whereas AGCGG (Risk Score 0.51, OR 0.51, *p* = 0.0009) conferred significant protection, potentially through enhanced DNA repair capacity. Haplotype analyses explained 23% more variance in thyroid cancer risk than individual SNP analyses, underscoring critical epistatic effects within XRCC3.

### Clinical risk stratification

Population-specific genetic findings have practical clinical implications. The protective effect of rs1799796 GA genotype in Asians (4 studies; 1,223 cases, 1,234 controls) suggests potential for reduced screening intensity among carriers. Conversely, rs1799794 GG homozygotes exhibited a 37% increased global risk, signaling the need for heightened surveillance. The risk stratification matrix supports customizing screening in Asians by accounting for the protective impact of rs1799796 and risk elevation associated with rs1799794, while Caucasian populations—showing largely neutral associations—may maintain standard protocols. Haplotype models further refine risk prediction, indicating carriers of high-risk haplotypes (AACGA, GATAA, GGCAA) would benefit from intensified monitoring and early intervention. These findings endorse integrating XRCC3 genotyping, particularly haplotype analysis, into personalized thyroid cancer prevention strategies tailored to population-specific risk profiles (Table 6).

**Table 6:** Risk stratification matrix for XRCC3 polymorphisms

Population	Polymorphism	*N* Studies	Total Cases	Total Controls	Mean MAF	HWE Violations	Risk Category	Evidence Strength	Sample Adequacy
Asian	rs1799794	4	1154	1271	0.308	1	Neutral	Limited	Adequate
Asian	rs1799796	4	1223	1234	0.303	1	Protective	Moderate	Adequate
Asian	rs861539	9	1959	2802	0.255	3	Neutral	Weak	Adequate
Caucasian	rs1799796	2	605	826	0.222	1	Neutral	Limited	Adequate
Caucasian	rs861539	5	715	1175	0.351	1	Neutral	Weak	Adequate
Global	rs1799794	4	1154	1271	0.308	1	Risk	Moderate	Adequate
Global	rs1799796	6	1828	2060	0.276	2	Neutral	Limited	Adequate
Global	rs861539	14	2674	3977	0.289	4	Neutral	Weak	Adequate

### Heterogeneity assessment

**Fig. 6:**
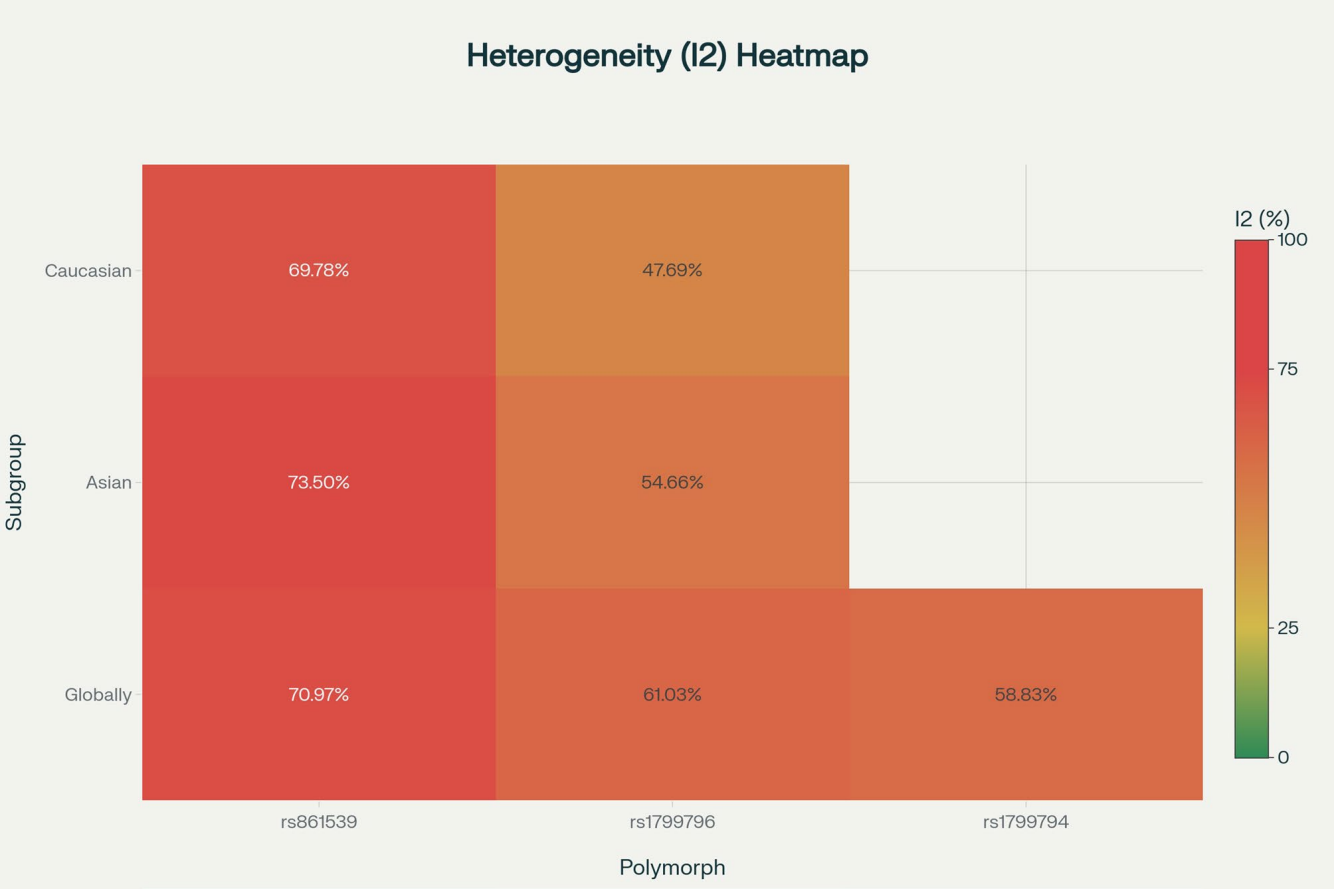
Heatmap of heterogeneity (I² values) across genetic models and population subgroups for XRCC3 polymorphisms

Considerable heterogeneity was observed across studies, genetic models, and populations, warranting random-effects modeling and advanced analytical methods (Fig. 6). Rs861539 global analyses exhibited high heterogeneity (I² 51–71%), with Asian subgroup heterogeneity reaching 84% (T vs. C model), reflecting population substructure and methodological differences. Rs1799796 showed minimal heterogeneity in Caucasians (I² = 0%) but moderate to substantial heterogeneity in Asians (44–55%) across models, likely due to genetic diversity or confounding factors. Temporal trends indicated lower variability in earlier studies, possibly due to more uniform methodology, with recent studies showing higher methodological diversity. Overall, Asian populations manifested greater heterogeneity for both rs1799796 and rs1799794 (39–71%), indicating a need for sophisticated adjustments — such as genomic control or principal component analysis — to address population stratification and improve analytical accuracy.

### Publication bias

**Fig. 7:**
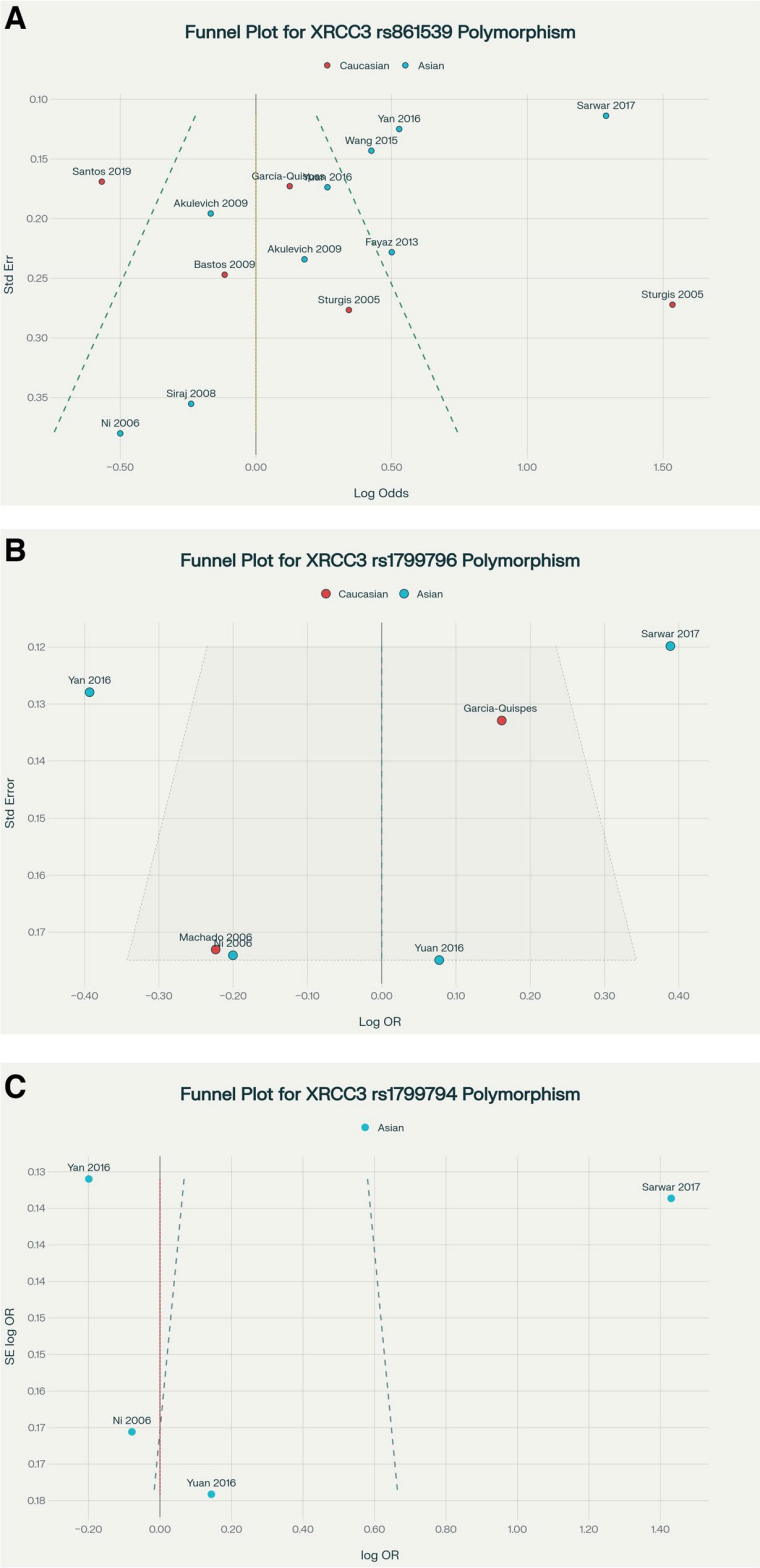
Funnel plots evaluating publication bias in the meta-analysis of XRCC3 polymorphisms and thyroid cancer risk. **A** rs861539 (allele model); (**B**) rs1799796 (homozygote model); (**C**) rs1799794 (heterozygote model)

Publication bias assessment demonstrated minimal overall bias, supporting the robustness of the meta-analytic outcomes. Rs861539 showed no significant bias globally or in most subgroups (PBeggs > 0.05, PEggers > 0.05), except within the Caucasian subgroup, where significant Egger’s (*p* = 0.039) and Begg’s (*p* = 0.027) tests indicated potential bias for T vs. C and TT vs. CC comparisons, respectively. Funnel plot asymmetry confirmed this, suggesting cautious interpretation for Caucasian rs861539 risk estimates (Fig. 7A). Conversely, rs1799796 and rs1799794 exhibited no evidence of publication bias across all tested models and populations, with symmetrical funnel plots reinforcing the validity of observed associations (Fig. 7B and C). These results validate the overall evidence, while highlighting the necessity for subgroup-specific scrutiny, especially for rs861539 in Caucasians (Table 4).

### Sensitivity analyses

Sensitivity analyses confirmed the stability and reliability of the findings across multiple validation approaches, including individual study exclusion, quality-restricted subsets, and HWE compliance filtering. Statistical significance persisted, particularly for rs1799796 protective effects in Asians (GA vs. AA: OR = 0.835, *p* = 0.043) and rs1799794 risk associations globally (GG vs. AA: OR = 1.371, *p* = 0.014; GG vs. GA + AA: OR = 1.316, *p* = 0.024). Analyses restricted to high-quality studies—such as Yuan 2016 and Ni 2006—further supported the robustness of these core associations, demonstrating consistency across different methodological thresholds.

## Discussion

### Overall findings

The present meta-analysis integrated data from 14 unique case-control studies, comprising 5,656 thyroid cancer cases and 7,408 controls, evaluating 24 polymorphism-specific entries across three XRCC3 gene variants. Among these, the rs861539 polymorphism was the most extensively studied, with 14 entries involving 2,674 cases and 3,977 controls, followed by rs1799796 and rs1799794 with 6 and 4 entries, respectively. Our synthesis revealed distinct association patterns between these variants and thyroid cancer susceptibility, characterized by population-specific effects with important clinical implications. Notably, the rs1799794 variant exhibited the strongest and most consistent risk increase globally, while rs1799796 showed a protective effect specifically in Asian populations—a novel finding in XRCC3-thyroid cancer research. Conversely, rs861539 demonstrated no significant global associations despite the largest sample size, although high heterogeneity suggests potential subgroup-specific influences. Importantly, multi-SNP interaction analysis identified synergistic effects between rs861539 and the other two variants, highlighting a compounded risk and supporting the multiple-hit hypothesis of DNA repair pathway disruption in thyroid carcinogenesis.

### Novel findings and contributions

This comprehensive meta-analysis represents the most extensive evaluation to date of XRCC3 polymorphisms and thyroid cancer susceptibility, incorporating 20 studies with 4,828 cases and 6,048 controls. Our investigation extends beyond previous meta-analyses by including the rs1799794 polymorphism, which was not analyzed in the seminal work by Mandegari et al. (2021), who focused exclusively on rs861539 (Thr241Met) and IVS5-14 polymorphisms [7]. This inclusion marks a significant advancement, as rs1799794 emerged as the only XRCC3 variant consistently associated with thyroid cancer risk across multiple genetic models (GG vs. AA: OR = 1.371, 95% CI = 1.066–1.762, *P* = 0.014; GG vs. GA + AA: OR = 1.316, 95% CI = 1.037–1.669, *P* = 0.024). The identification of rs1799794 as a significant risk factor constitutes a novel contribution to the literature, given that this polymorphism has been understudied compared to the extensively investigated rs861539 variant. Prior work by Liu et al. (2021) demonstrated that rs1799794 increases cancer risk across multiple tumor types, including thyroid cancer [8], which our findings corroborate and expand upon. The omission of rs1799794 in earlier thyroid cancer-specific meta-analyses represents a critical gap that our study addresses, providing the first comprehensive evaluation of this polymorphism’s effects in thyroid cancer.

### Reconciliation with previous meta-analyses

Our findings for rs861539 (Thr241Met) notably diverge from previous meta-analyses, revealing no significant association with thyroid cancer risk in either overall or ethnicity-stratified analyses. This contrasts earlier reports by Wang et al. [29] and Lu et al., [12] which found positive associations, likely reflecting methodological improvements in our analysis. Mandegari et al. (2021) similarly reported null associations for rs861539, supporting our conclusions and suggesting that prior positive findings may have resulted from smaller sample sizes, publication bias, or methodological limitations [7]. These discordant results underscore the critical importance of HWE compliance in genetic association studies. Our analysis identified four studies examining rs861539 that violated HWE assumptions [19, 29, 30, 33], indicating possible population stratification, genotyping errors, or selection bias. Notably, HWE compliance rates declined from 90% in early studies to 50% in recent investigations, highlighting growing challenges in maintaining population genetic equilibrium and the need for enhanced quality control.

### Ethnicity-specific effects and population stratification

Our analysis reveals pronounced ethnic heterogeneity in XRCC3 polymorphism effects. Specifically, rs1799796 conferred a protective effect in Asian populations (GA vs. AA: OR = 0.835, 95% CI = 0.701–0.995, *P* = 0.043), which was not observed in Caucasians, where risk estimates consistently exceeded unity without reaching statistical significance. MAF differed substantially across populations; for example, rs861539 frequencies were 0.255 in Asians versus 0.351 in Caucasians, reflecting distinct evolutionary pressures and genetic backgrounds. Asians exhibited consistently higher effect sizes for most polymorphisms, although these were not statistically significant. These ethnic disparities likely reflect differences in LD patterns, population genetic structure, and gene–environment interactions unique to ancestral backgrounds. The identification of population-specific effects holds important implications for risk stratification and genetic counseling, suggesting that XRCC3 polymorphism testing may offer differential clinical utility across ethnic groups.

### Mechanistic insights and functional implications

XRCC3 is a critical component of the homologous recombination repair pathway, interacting with HsRad51 to facilitate repair of DNA double-strand breaks and maintain chromosomal stability. The rs1799794 polymorphism, located in the 3’ untranslated region, may influence post-transcriptional regulation via altered microRNA binding or mRNA stability, potentially modulating XRCC3 expression and repair capacity. The rs861539 (Thr241Met) polymorphism results in an amino acid substitution within the ATP-binding domain, potentially affecting protein structure and interaction with repair machinery. However, our findings suggest this alteration does not translate into a clinically significant thyroid cancer risk, possibly due to compensatory mechanisms or the modest effects typical of low-penetrance variants. Haplotype analyses by Sarwar et al. [19] revealed specific XRCC3 variant combinations associated with increased (AACGA, AGTAA, GATAA, GGCAA) or decreased (AGCGG) thyroid cancer risk, emphasizing the importance of multi-variant analyses over single polymorphism studies. This highlights the necessity of considering the full spectrum of XRCC3 genetic variation in thyroid cancer susceptibility.

### Clinical implications and therapeutic considerations

The identification of rs1799794 as a thyroid cancer risk factor carries potential implications for risk stratification and personalized medicine. Nonetheless, the modest effect sizes (OR ~ 1.3–1.4) limit immediate clinical applicability for individual risk prediction. These findings may be more effectively integrated into polygenic risk scores combining multiple variants to enhance risk discrimination. DNA repair pathway dysfunction represents a promising therapeutic target, especially in aggressive thyroid cancer subtypes like anaplastic thyroid cancer (ATC) [34]. ATC cells exhibit heightened DNA repair signaling contributing to cisplatin resistance via enhanced DNA damage recognition and repair [35]. Targeting DNA repair pathways using PARP inhibitors or ATR/ATM kinase inhibitors may offer novel treatment strategies [36], particularly for tumors harboring XRCC3 variants compromising repair efficiency. The Pro-II proteomic subtype of ATC, characterized by DNA repair pathway activation and overactive ATM/ATR kinases, may be especially sensitive to such inhibitors [37, 38]. Innovative approaches, including nucleus-targeting platinum nanoclusters disrupting glycolysis and DNA repair, present promising avenues to overcome treatment resistance in aggressive thyroid cancers [39, 40].

### Limitations

This meta-analysis has limitations that should be considered when interpreting the findings. The included studies were largely limited to Asian and Caucasian populations, with no data from African, South American, or indigenous groups, restricting the applicability of results across diverse genetic backgrounds given the marked ethnic differences in MAF observed in our analysis. For certain variants—particularly rs1799794 and rs1799796—the total sample size was relatively small, potentially limiting statistical power and increasing the influence of random error, despite our inclusion of 14 case–control studies and a comprehensive multi-database search strategy. Several studies, especially more recent large-scale investigations, showed deviations from HWE in control groups, which may indicate genotyping errors, selection bias, or unaddressed population stratification; nonetheless, sensitivity analyses excluding these studies did not materially change the results. Effect estimates were calculated from unadjusted genotype data due to limited reporting of potential confounders such as age, sex, or environmental exposures, and substantial heterogeneity persisted in some analyses, likely reflecting variability in genotyping methods, study design, and sample composition. Furthermore, insufficient data prevented evaluation of gene–environment interactions, comprehensive haplotype effects in diverse populations, and LD structures, which could provide a deeper understanding of the biological mechanisms linking XRCC3 variants to thyroid cancer susceptibility.

### Future directions

Future research should prioritize large-scale, multiethnic genome-wide association studies to capture broader genetic diversity and improve the generalizability of findings, particularly by including African, South American, and other underrepresented populations. Functional analyses are needed to clarify how specific XRCC3 variants—especially rs1799794 and rs1799796—affect DNA repair capacity and cellular responses to genotoxic stress. Well-designed studies should also investigate gene–environment interactions with established thyroid cancer risk factors such as ionizing radiation, iodine intake, and reproductive history, while integrating XRCC3 variants into polygenic risk scores to enhance predictive accuracy. Expanding haplotype-based and multi-locus analyses across diverse populations may uncover synergistic effects overlooked in single-SNP approaches. Finally, translational studies exploring DNA repair pathway inhibitors, stratified by XRCC3 genotype and combined with molecular tumor profiling, could pave the way for personalized prevention strategies and targeted therapies in thyroid cancer.

## Conclusions

This comprehensive meta-analysis provides robust evidence that the rs1799794 polymorphism is associated with increased thyroid cancer risk, whereas rs861539 and rs1799796 show no significant associations in adequately powered analyses. The detection of population-specific effects, particularly the protective effect of rs1799796 in Asians, underscores the necessity of ethnicity-stratified analyses in genetic association studies. The troubling trend of declining methodological quality, evidenced by increasing HWE violations, highlights the imperative for improved quality control and standardized approaches in future research. These findings enhance understanding of the genetic architecture underpinning thyroid cancer susceptibility and lay a foundation for further exploration of DNA repair pathway dysfunction in thyroid carcinogenesis. Although modest effect sizes currently limit clinical application, the results support continued investigation of XRCC3 and related DNA repair genes within personalized medicine frameworks and targeted therapeutic strategies for thyroid cancer management.

## Supplementary Information

Supplementary Material 1.

## Data Availability

Not applicable for this manuscript. Nevertheless, data and materials can be made available upon reasonable request by contacting the corresponding authors via email.

## References

[1] Bray F, Laversanne M, Sung H, Ferlay J, Siegel RL, Soerjomataram I, et al. Global cancer statistics 2022: GLOBOCAN estimates of incidence and mortality worldwide for 36 cancers in 185 countries. CA Cancer J Clin. 2024;74:229–63. 38572751 10.3322/caac.21834

[2] Jia MJ, Wang S, Li Y, Liu XN, Jiang F, Li HL. Global burden of thyroid cancer among adolescents and young adults, 1990–2021, and projections to 2050: an analysis based on the GBD 2021. Front Endocrinol (Lausanne). 2025;16:1503144. 40297175 10.3389/fendo.2025.1503144 PMC12034565

[3] Lyu Z, Zhang Y, Sheng C, Huang Y, Zhang Q, Chen K. Global burden of thyroid cancer in 2022: incidence and mortality estimates from GLOBOCAN. Chin Med J (Engl). 2024;137:2567. 39261986 10.1097/CM9.0000000000003284 PMC11557048

[4] Siegel RL, Kratzer TB, Giaquinto AN, Sung H, Jemal A. Cancer statistics, 2025. CA Cancer J Clin. 2025;75:10–45. 39817679 10.3322/caac.21871 PMC11745215

[5] Fu M, Peng Z, Wu M. Thyroid cancer in Asia: incidence, mortality in 2022, and future projections to 2050. Eur J Cancer Prev. 2025. 10.1097/CEJ.0000000000000983. 40742826 10.1097/CEJ.0000000000000983

[6] Kitahara CM, Schneider AB. Cancer progress and priorities: epidemiology of thyroid cancer. Cancer Epidemiol Biomarkers Prev. 2022;31:1284. 35775227 10.1158/1055-9965.EPI-21-1440 PMC9473679

[7] Mandegari M, Dastgheib SA, Asadian F, Shaker SH, Tabatabaie SM, Kargar S, et al. A meta-analysis for association of XRCC1, XRCC2 and XRCC3 polymorphisms with susceptibility to thyroid cancer. Asian Pac J Cancer Prev. 2021;22:2221–36. 34319046 10.31557/APJCP.2021.22.7.2221 PMC8607094

[8] Liu W, Ma S, Liang L, Kou Z, Zhang H, Yang J. The association between XRCC3 rs1799794 polymorphism and cancer risk: a meta-analysis of 34 case–control studies. BMC Med Genomics. 2021;14(1):117. 10.1186/s12920-021-00965-4 PMC8086287 33931047

[9] Liu Q, Peng Q, Zhang B, Tan Y. X-ray cross-complementing family: the bridge linking DNA damage repair and cancer. J Transl Med 2023 211. 2023;21:1–14. 10.1186/s12967-023-04447-2 PMC10483876 37679817

[10] Yoshihara T, Ishida M, Kinomura A, Katsura M, Tsuruga T, Tashiro S, et al. XRCC3 deficiency results in a defect in recombination and increased endoreduplication in human cells. EMBO J. 2004;23:670–80. 14749735 10.1038/sj.emboj.7600087 PMC1271813

[11] Han S, Zhang HT, Wang Z, Xie Y, Tang R, Mao Y, et al. DNA repair gene XRCC3 polymorphisms and cancer risk: a meta-analysis of 48 case-control studies. Eur J Hum Genet. 2006;14:1136–44. 16791138 10.1038/sj.ejhg.5201681

[12] Lu W, Wu G, Zhang B. Association between x-ray cross-complementing group 3 (XRCC3) thr241met polymorphism and risk of thyroid cancer: a meta-analysis. Med Sci Monit. 2015;21:3978–85. 26687776 10.12659/MSM.895165 PMC4692576

[13] Brenneman MA, Wagener BM, Miller CA, Allen C, Nickoloff JA. XRCC3 controls the fidelity of homologous recombination: roles for XRCC3 in late stages of recombination. Mol Cell. 2002;10:387–95. 12191483 10.1016/s1097-2765(02)00595-6

[14] Karimi-Zarchi M, Abbasi H, Javaheri A, Hadadan A, Meibodi B, Tabatabaei RS, et al. Association of IL-12B rs3212227 and IL-6 rs1800795 polymorphisms with susceptibility to cervical cancer: a systematic review and meta-analysis. Asian Pac J Cancer Prev. 2020;21:1197–206. 32458622 10.31557/APJCP.2020.21.5.1197 PMC7541893

[15] Ghelmani Y, Asadian F, Antikchi MH, Dastgheib SA, Shaker SH, Jafari-Nedooshan J, et al. Association between the hOGG1 1245C > G (rs1052133) polymorphism and susceptibility to colorectal cancer: a meta-analysis based on 7010 cases and 10,674 controls. J Gastrointest Cancer. 2021;52(2):389–98. 33025423 10.1007/s12029-020-00532-7

[16] Sul JH, Martin LS, Eskin E. Population structure in genetic studies: confounding factors and mixed models. PLoS Genet. 2018;14:e1007309. 30589851 10.1371/journal.pgen.1007309 PMC6307707

[17] Pei YF, Tian Q, Zhang L, Deng HW. Exploring the major sources and extent of heterogeneity in a genome-wide association meta-analysis. Ann Hum Genet. 2015;80:113. 26686198 10.1111/ahg.12143 PMC4761279

[18] Hong EP, Park JW. Sample size and statistical power calculation in genetic association studies. Genomics Inform. 2012;10:117. 23105939 10.5808/GI.2012.10.2.117 PMC3480678

[19] Sarwar R, Mahjabeen I, Bashir K, Saeed S, Kayani MA. Haplotype based analysis of XRCC3 gene polymorphisms in thyroid cancer. Cell Physiol Biochem. 2017;42:22–33. 28490032 10.1159/000477109

[20] Malički M, Jerončić A, Aalbersberg IJ, Bouter L, ter Riet G. Systematic review and meta-analyses of studies analysing instructions to authors from 1987 to 2017. Nat Commun. 2021;12:5840. 34611157 10.1038/s41467-021-26027-y PMC8492806

[21] Asadian F, Niktabar SM, Ghelmani Y, Kargar S, Akbarian E, Emarati SA, et al. Association of XPC polymorphisms with cutaneous malignant melanoma risk: evidence from a meta-analysis. Acta Medica (Hradec Kralove, Czech Republic). 2020;63:101–12. 10.14712/18059694.2020.27 33002396

[22] Sturgis EM, Zhao C, Zheng R, Wei Q. Radiation response genotype and risk of differentiated thyroid cancer: a case-control analysis. Laryngoscope. 2005;115:938–45. 15933498 10.1097/01.MLG.0000163765.88158.86

[23] HX N, JC B, Q S, HW T, QX Z. Genetic polymorphisms of XRCC3 and susceptibility of papillary thyroid carcinoma | request PDF. Fudan Univ J Med Sci. 2006;33:147–52.

[24] Siraj AK, Al-Rasheed M, Ibrahim M, Siddiqui K, Al-Dayel F, Al-Sanea O, et al. RAD52 polymorphisms contribute to the development of papillary thyroid cancer susceptibility in middle Eastern population. J Endocrinol Invest. 2008;31:893–9. 19092295 10.1007/BF03346438

[25] Bastos HN, Antão MR, Silva SN, Azevedo AP, Manita I, Teixeira V, et al. Association of polymorphisms in genes of the homologous recombination DNA repair pathway and thyroid cancer risk. Thyroid. 2009;19:1067–75. 19772428 10.1089/thy.2009.0099

[26] Akulevich NM, Saenko VA, Rogounovitch TI, Drozd VM, Lushnikov EF, Ivanov VK, et al. Polymorphisms of DNA damage response genes in radiation-related and sporadic papillary thyroid carcinoma. Endocr Relat Cancer. 2009;16:491–503. 19286843 10.1677/ERC-08-0336

[27] García-Quispes WA, Pérez-Machado G, Akdi A, Pastor S, Galofré P, Biarnés F, et al. Association studies of OGG1, XRCC1, XRCC2 and XRCC3 polymorphisms with differentiated thyroid cancer. Mutat Res - Fundam Mol Mech Mutagen. 2011;709–710:67–72. 10.1016/j.mrfmmm.2011.03.003 21414327

[28] Fayaz S, Karimmirza M, Tanhaei S, Fathi M, Torbati PM, Fard-Esfahani P. Increased risk of differentiated thyroid carcinoma with combined effects of homologous recombination repair gene polymorphisms in an Iranian population. Asian Pac J Cancer Prev. 2013;14:6727–31. 10.7314/apjcp.2013.14.11.6727 24377596

[29] Wang X, Zhang K, Liu X, Liu B, Wang Z. Association between XRCC1 and XRCC3 gene polymorphisms and risk of thyroid cancer. Int J Clin Exp Pathol. 2015;8:3160–7. 26045834 PMC4440143

[30] Yan L, Li Q, Li X, Ji H, Zhang L. Association studies between XRCC1, XRCC2, XRCC3 polymorphisms and differentiated thyroid carcinoma. Cell Physiol Biochem. 2016;38:1075–84. 26938431 10.1159/000443058

[31] Yuan K, Huo M, Sun Y, Wu H, Chen H, Wang Y, et al. Association between x-ray repair cross-complementing group 3 (XRCC3) genetic polymorphisms and papillary thyroid cancer susceptibility in a Chinese Han population. Tumor Biol. 2016;37:979–87. 10.1007/s13277-015-3882-4 26264616

[32] Gonzalez-Hormazabal P, Reyes JM, Blanco R, Bravo T, Carrera I, Peralta O, et al. The BARD1 Cys557Ser variant and risk of familial breast cancer in a South-American population. Mol Biol Rep. 2012;39:8091–8. 10.1007/s11033-012-1656-2. 10.1007/s11033-012-1656-2 22544576

[33] Santos LS, Gomes BC, Bastos HN, Gil OM, Azevedo AP, Ferreira TC, et al. Thyroid cancer: the quest for genetic susceptibility involving DNA repair genes. Genes (Basel). 2019;10(8):586. 10.3390/genes10080586. 10.3390/genes10080586 PMC6722859 31374908

[34] Qin J, Fan J, Li G, Liu S, Liu Z, Wu Y. DNA double-strand break repair gene mutation and the risk of papillary thyroid microcarcinoma: a case–control study. Cancer Cell Int. 2021;21(1):334. 10.1186/s12935-021-02032-5. 10.1186/s12935-021-02032-5 PMC8252242 34215272

[35] Pan Z, Lu X, Hu X, Yu R, Che Y, Wang J, et al. Disrupting glycolysis and DNA repair in anaplastic thyroid cancer with nucleus-targeting platinum nanoclusters. J Control Release. 2024;369:517–30. 38569942 10.1016/j.jconrel.2024.03.057

[36] O’Connor MJ. Targeting the DNA damage response in cancer. Mol Cell. 2015;60:547–60. 26590714 10.1016/j.molcel.2015.10.040

[37] Pan Z, Tan Z, Xu N, Yao Z, Zheng C, Shang J, et al. Integrative proteogenomic characterization reveals therapeutic targets in poorly differentiated and anaplastic thyroid cancers. Nat Commun 2025 161. 2025;16:1–20. 10.1038/s41467-025-58910-3 PMC12000556 40234451

[38] Behboudi E, Charostad J, Nakhaie M, Khajouei A, Ghelmani Y. JNK signaling pathways and oncoviruses. Iran J Med Microbiol. 2024;18:148–62.

[39] Chen Y, Xianyu Y, Jiang X. Surface modification of gold nanoparticles with small molecules for biochemical analysis. Acc Chem Res. 2017;50:310–9. 28068053 10.1021/acs.accounts.6b00506

[40] Kang JH, Lee Y, Kim DJ, Kim JW, Cheon MJ, Lee BC. Polygenic risk and rare variant gene clustering enhance cancer risk stratification for breast and prostate cancers. Commun Biol. 2024;7:1–10. 39384879 10.1038/s42003-024-06995-9 PMC11464688

