# Supplementary material for: A comprehensive compilation of data on the association between XRCC3 polymorphisms and thyroid cancer susceptibility
Source: BMC Endocr Disord. 2025 Oct 16;25:231. doi: 10.1186/s12902-025-02044-6 (PMC12529809; doi:10.1186/s12902-025-02044-6)
Supplement: Supplementary file 1 — Supplementary Material 1. [file 12902_2025_2044_MOESM1_ESM.docx]

| **Section and Topic** | **Item #** | **Checklist item** | **Location where item is reported** |  |  |
| --- | --- | --- | --- | --- | --- |
| **TITLE** | | |  |  |  |
| Title | 1 | Identify the report as a systematic review. | Page 1 - Title clearly identifies this as a "comprehensive compilation" and meta-analysis |  |  |
| **ABSTRACT** | | |  |  |  |
| Abstract | 2 | See the PRISMA 2020 for Abstracts checklist. | Page 1 - Structured abstract with Background, Methods, Results, and Conclusions |  |  |
| **INTRODUCTION** | | |  |  |  |
| Rationale | 3 | Describe the rationale for the review in the context of existing knowledge. | Pages 1-3 - Introduction section describes thyroid cancer burden, DNA repair mechanisms, and inconsistent findings |  |  |
| Objectives | 4 | Provide an explicit statement of the objective(s) or question(s) the review addresses. | Page 3 - "This meta-analysis aimed to clarify the associations between XRCC3 polymorphisms and thyroid cancer risk" |  |  |
| **METHODS** | | |  |  |  |
| Eligibility criteria | 5 | Specify the inclusion and exclusion criteria for the review and how studies were grouped for the syntheses. | Page 4 - "Inclusion and Exclusion Criteria" section |  |  |
| Information sources | 6 | Specify all databases, registers, websites, organisations, reference lists and other sources searched or consulted to identify studies. Specify the date when each source was last searched or consulted. | Page 3-4 - "Publication Search" section lists 25+ databases searched up to July 10, 2025 |  |  |
| Search strategy | 7 | Present the full search strategies for all databases, registers and websites, including any filters and limits used. | Page 4 - Search strategy using MeSH terms and keywords described |  |  |
| Selection process | 8 | Specify the methods used to decide whether a study met the inclusion criteria of the review, including how many reviewers screened each record and each report retrieved, whether they worked independently, and if applicable, details of automation tools used in the process. | Page 4 - "two reviewers" conducted independent searches and screening |  |  |
| Data collection process | 9 | Specify the methods used to collect data from reports, including how many reviewers collected data from each report, whether they worked independently, any processes for obtaining or confirming data from study investigators, and if applicable, details of automation tools used in the process. | Page 5 - "Data extraction was conducted independently by two researchers using a standardized approach" |  |  |
| Data items | 10a | List and define all outcomes for which data were sought. Specify whether all results that were compatible with each outcome domain in each study were sought (e.g. for all measures, time points, analyses), and if not, the methods used to decide which results to collect. | Page 5 - Lists extracted data including genotype distributions, MAF, HWE status |  |  |
|  | 10b | List and define all other variables for which data were sought (e.g. participant and intervention characteristics, funding sources). Describe any assumptions made about any missing or unclear information. | Page 5 - Extracted data includes author, year, ethnicity, country, genotyping methods, sample sizes |  |  |
| Study risk of bias assessment | 11 | Specify the methods used to assess risk of bias in the included studies, including details of the tool(s) used, how many reviewers assessed each study and whether they worked independently, and if applicable, details of automation tools used in the process. | Page 6 - Quality assessment described with scores 2.0-6.0 based on sample size, HWE adherence, control matching |  |  |
| Effect measures | 12 | Specify for each outcome the effect measure(s) (e.g. risk ratio, mean difference) used in the synthesis or presentation of results. | Page 5 - "pooled odds ratios (ORs) with 95% confidence intervals (CIs)" under five genetic models |  |  |
| Synthesis methods | 13a | Describe the processes used to decide which studies were eligible for each synthesis (e.g. tabulating the study intervention characteristics and comparing against the planned groups for each synthesis (item #5)). | Page 4-5 - Inclusion/exclusion criteria and data extraction process |  |  |
|  | 13b | Describe any methods required to prepare the data for presentation or synthesis, such as handling of missing summary statistics, or data conversions. | Page 5 - Authors contacted for missing data |  |  |
|  | 13c | Describe any methods used to tabulate or visually display results of individual studies and syntheses. | Tables 1-6, Figures 1-7 referenced throughout |  |  |
|  | 13d | Describe any methods used to synthesize results and provide a rationale for the choice(s). If meta-analysis was performed, describe the model(s), method(s) to identify the presence and extent of statistical heterogeneity, and software package(s) used. | Page 5 - Fixed/random effects models, I² statistic, CMA software version 2.0 |  |  |
|  | 13e | Describe any methods used to explore possible causes of heterogeneity among study results (e.g. subgroup analysis, meta-regression). | Page 5 - "Subgroup analyses by ethnicity, genotyping method, and study quality" |  |  |
|  | 13f | Describe any sensitivity analyses conducted to assess robustness of the synthesized results. | Page 5 - "Sensitivity analyses by sequentially excluding individual studies and HWE-violating studies" |  |  |
| Reporting bias assessment | 14 | Describe any methods used to assess risk of bias due to missing results in a synthesis (arising from reporting biases). | Page 5 - "Begg's funnel plots and Egger's regression test" |  |  |
| Certainty assessment | 15 | Describe any methods used to assess certainty (or confidence) in the body of evidence for an outcome. | Page 6-8 - Quality assessment and HWE compliance evaluation |  |  |
| **RESULTS** | | |  |  |  |
| Study selection | 16a | Describe the results of the search and selection process, from the number of records identified in the search to the number of studies included in the review, ideally using a flow diagram. | Page 6 - "498 potentially relevant articles... 14 unique case-control studies" (Figure 1 referenced) |  |  |
|  | 16b | Cite studies that might appear to meet the inclusion criteria, but which were excluded, and explain why they were excluded. | Page 6 - "79 were excluded for irrelevance or insufficient data" |  |  |
| Study characteristics | 17 | Cite each included study and present its characteristics. | Page 6, Table 1 - Detailed study characteristics presented |  |  |
| Risk of bias in studies | 18 | Present assessments of risk of bias for each included study. | Pages 6-7 - Quality scores and HWE violations discussed |  |  |
| Results of individual studies | 19 | For all outcomes, present, for each study: (a) summary statistics for each group (where appropriate) and (b) an effect estimate and its precision (e.g. confidence/credible interval), ideally using structured tables or plots. | Tables 4-5, Figures 4-7 - Individual study results and pooled estimates |  |  |
| Results of syntheses | 20a | For each synthesis, briefly summarise the characteristics and risk of bias among contributing studies. | Pages 7-9 - Population-specific analyses and quality assessment |  |  |
|  | 20b | Present results of all statistical syntheses conducted. If meta-analysis was done, present for each the summary estimate and its precision (e.g. confidence/credible interval) and measures of statistical heterogeneity. If comparing groups, describe the direction of the effect. | Pages 8-9, Tables 4-5 - Pooled ORs with 95% CIs and I² values |  |  |
|  | 20c | Present results of all investigations of possible causes of heterogeneity among study results. | Pages 9-10 - Subgroup analyses by ethnicity and temporal trends |  |  |
|  | 20d | Present results of all sensitivity analyses conducted to assess the robustness of the synthesized results. | Page 11 - "Sensitivity analyses confirmed stability and reliability" |  |  |
| Reporting biases | 21 | Present assessments of risk of bias due to missing results (arising from reporting biases) for each synthesis assessed. | Page 10-11 - Begg's and Egger's tests results, Figure 7 |  |  |
| Certainty of evidence | 22 | Present assessments of certainty (or confidence) in the body of evidence for each outcome assessed. | Pages 11-12 - Discussion of study quality and limitations |  |  |
| **DISCUSSION** | | |  |  |  |
| Discussion | 23a | Provide a general interpretation of the results in the context of other evidence. | Pages 11-14 - "Overall Findings" and comparison with previous meta-analyses |  |  |
|  | 23b | Discuss any limitations of the evidence included in the review. | Page 16 - "Limitations" section |  |  |
|  | 23c | Discuss any limitations of the review processes used. | Page 16 - Discussion of HWE violations, sample size limitations |  |  |
|  | 23d | Discuss implications of the results for practice, policy, and future research. | Pages 14-17 - "Clinical Implications," "Future Directions," and "Conclusions" |  |  |
| **OTHER INFORMATION** | | |  |  |  |
| Registration and protocol | 24a | Provide registration information for the review, including register name and registration number, or state that the review was not registered. | Not registered |  |  |
|  | 24b | Indicate where the review protocol can be accessed, or state that a protocol was not prepared. | Not registered |  |  |
|  | 24c | Describe and explain any amendments to information provided at registration or in the protocol. | Not registered |  |  |
| Support | 25 | Describe sources of financial or non-financial support for the review, and the role of the funders or sponsors in the review. | Page 17 - "Funding: No funding source was disclosed" |  |  |
| Competing interests | 26 | Declare any competing interests of review authors. | Page 17 - "Conflicts of Interest: The authors declare no conflicts of interest" |  |  |
| Availability of data, code and other materials | 27 | Report which of the following are publicly available and where they can be found: template data collection forms; data extracted from included studies; data used for all analyses; analytic code; any other materials used in the review. | Page 17 - "data and materials can be made available upon reasonable request" |  |  |

*From:*  Page MJ, McKenzie JE, Bossuyt PM, Boutron I, Hoffmann TC, Mulrow CD, et al. The PRISMA 2020 statement: an updated guideline for reporting systematic reviews. BMJ 2021;372:n71. doi: 10.1136/bmj.n71. This work is licensed under CC BY 4.0. To view a copy of this license, visit <https://creativecommons.org/licenses/by/4.0/>
